# Organic and conventional alternative curing ingredients effects on quality and sensory attributes of deli-style Turkey

**DOI:** 10.1016/j.psj.2025.105370

**Published:** 2025-05-28

**Authors:** Siyuan Sheng, Erin M. Silva, Steven C. Ricke, James R. Claus

**Affiliations:** a Meat Science and Animal Biologics Discovery, Department of Animal & Dairy Sciences, University of Wisconsin-Madison, Madison, Wisconsin, United States; b Department of Plant Pathology, University of Wisconsin-Madison, Madison, Wisconsin 53706, United States

**Keywords:** Turkey, Sensory, GC-MS/MS, Organic processed meats, Alternative curing

## Abstract

Deli-style turkey breast products were cured with sodium nitrite (SN), pre-converted conventional grown celery (CEL), Swiss Chard (SC), organic grown celery (OCEL), and organic Swiss Chard (OSW) with an ingoing sodium nitrite equivalent of 150 ppm and salt content of 1.60 %. Cured and total meat pigments, total myoglobin content, and salt content were assessed on day 0; color, pH, and residual nitrite were evaluated on days 0, 15, 30, 45, and 60. A 24-h color depletion study was conducted on all treatments (TRTs) after storage (day 15). Consumer panelists were recruited to evaluate sensory attributes of all TRTs. During the storage and light display, SN exhibited the lightest color and the lowest (*P* < 0.05) levels of yellowness. At 0 h, Celery TRTs demonstrated lower (*P* < 0.05) hue angle compared to Swiss chard TRTs. This color difference gradually becomes unnoticeable by the conclusion of the test. Furthermore, the choice between organic and non-organic curing does not affect the color of the finished products. Sensory results indicated that OSW exhibited greater earthiness (*P* < 0.05) than all other TRTs, except for CEL, which showed no significant difference (*P* > 0.05). Sensory evaluation results also indicated that OSW displayed the greatest non-meat aftertaste (*P* < 0.001) than all other TRTs and lower bitterness (*P* = 0.009) than SN. However, regardless of the different plant sources of nitrite (Celery or Swiss Chard) and growing practice of plant powder (conventional or organic), sensory evaluation found that these alternative cures were equivalent (*P* > 0.05) to SN in overall liking difference and relevant purchase intent. Volatile compounds (VOCs) analysis revealed a distinctively different distribution of VOCs among the different sources of nitrite. The presence of terpene alcohol and lower abundance of aldehydes in OSW could explain the findings identified in the sensory evaluation.

## Introduction

Nitrites play a crucial role in the curing process, contributing to food safety and quality. When added to meat, nitrites inhibit the growth of pathogenic bacteria, such as *Clostridium botulinum*, and prevent lipid peroxidation, thereby extending shelf life from both food safety and quality perspectives. Nitrite, when reduced to nitric oxide during meat processing, binds to iron in myoglobin. This interaction stabilizes the heme iron, preventing it from triggering lipid oxidation, a major factor in limiting meat quality and shelf life (Zhang et al., 2022). Without this stabilization, free iron, a powerful catalyst, would accelerate lipid oxidation, leading to product quality deterioration (Liu et al., 2023). Additionally, nitrites are responsible for the characteristic pink color and distinct flavor of cured meats, making them visually appealing and flavorful. While chemical sources of nitrites remain in use, alternative sources are being explored. Consequently, in the early 2000s, driven by increasing consumer demand for transparency and safety for food and food ingredients, clean label trends in the meat industry have gained increased interest (Delgado-Pando et al., 2021)

The shift towards natural curing agents aligns with consumer preferences for clean-label and organic products, while still presumably maintaining the desired quality attributes of cured meats. Traditionally, nitrites have been added in purified forms, but there is growing interest in natural sources, such as celery (*Apium graveolens* L.) powder, which contains naturally occurring nitrates that can be either converted to nitrites during the curing process or added into processed meats as a pre-converted form. In addition, cherry or acerola powder was found to be a good candidate to replace sodium erythorbate as a curing accelerator (Sebranek and Bacus, 2007). The U.S. Department of Agriculture (USDA) regulates meat products that use natural curing agents requiring them to be labelled as “Uncured” or “No Nitrate or Nitrite Added…”(USDA, 1979). In alignment with the clean label trend and consumer awareness of organic foods, the organic meat market is experiencing significant growth, driven by increasing health consciousness and concerns regarding over use of additives and chemicals (Grand View Research, 2023). The market size for organic meat products is projected to grow from USD 20.27 billion in 2024 to USD 29.71 billion by 2029, with a compound annual growth rate of 7.94 % (Mordor Intelligence, 2024).

From a regulatory perspective, purified sodium or potassium nitrite commonly used in the traditional meat industry are not permitted in organic products by the USDA National Organic Program (NOP), instead, celery powder has been used in the organic meat industry as a meat curing ingredient since the inception of NOP (USDA, 2024). The National Organic Standards Board (NOSB) currently permits the use of non-organic celery powder in organic products. However, the NOSB is in the process of conducting sunset reviews to transition to organic sources of celery powder. This involves evaluating whether non-organic celery powder should remain on the National List of Allowed and Prohibited Substances or be replaced with organic alternatives (NOSB, 2023). In addition to celery, Swiss chard (*Beta vulgaris* L. var. *cicla*) is a well-recognized alternative vegetable source for curing powders, owing to its high nitrate concentration (Calderón et al., 2025). In comparison to celery, Swiss chard reportedly imparts a less pronounced aftertaste and aromatic volatile compounds (Sheng et al., 2025a, 2025b) and is associated with fewer allergenicity concerns (Ballmer‐Weber et al., 2002).

Previous studies comparing celery-cured products with those cured using traditional purified nitrites have concluded that celery powder-cured meats offer similar quality attributes, including proximate composition, color, flavor, and sensory to those cured with purified nitrite (Alkazzaz et al., 2022; Jin et al., 2018; ORAL, 2023; Posthuma et al., 2018). However, it is unknown whether curing powder from organically grown celery or Swiss chard offers similar quality and sensory attributes to that from conventionally grown ones. Additionally, it remains unclear whether other organically grown vegetables can serve as a nitrite source in cured meats and provide comparable or enhanced quality and sensory attributes compared to synthetic sodium nitrite. Therefore, the purpose of this study was to assess the quality and sensory attributes of conventional and organic grown vegetable nitrite sourced alternative curing powder on Ready-to-Eat (RTE) deli-style turkey formulated with an equivalent amount of SN and salt (sodium chloride) at comparable industry-use levels. Furthermore, the study conducted comprehensive profiling of volatile compounds in various treatments of deli-style turkey to evaluate their correlation with the sensory attributes.

## Material and methods

### Experimental design

This study was conducted using quality analysis (physicochemical) and paired sensory and volatile compounds analysis to evaluate the effect of various sources of nitrite on deli-style turkey’s product quality and sensory attributes, employing five treatments (TRTs). The treatments included four vegetable pre-converted curing powders (celery powder, CEL; organic celery powder, OCEL; Swiss chard powder, SW; organic Swiss chard powder, OSW) and a control curing agent treatment (synthetic sodium nitrite, SN: added as a cure mix that containing 6.25 % sodium nitrite and 93.75 % sodium chloride). The non-meat ingredients were added at a total of 20.0 % of the meat block weight (Table 1). Boneless skinless turkey (9.07 kg meat block) was formulated to contain 150 ppm sodium nitrite equivalent and 1.60 % sodium chloride based on the meat block weight and on nitrite and salt concentration determined in curing powder. In addition, the turkey was formulated to contain 1.8 % modified corn starch (C*EmTex, Cargill Inc. Minnetonka, Minnesota, U.S.A.), 1.2 % dextrose, and 0.40 % sodium tripolyphosphate (w/w). Curing was accelerated with sodium erythorbate (547 ppm) for sodium nitrite (SN) TRT, and cherry powder for the vegetable source nitrite TRTs.

**Table 1:** Formulations^1^ used to produce Deli-Style Turkey with different alternative cure ingredients in addition to a standard sodium nitrite control.

Treatments^2^	Water (%)	Salt^3^ (%)	Curing ingredients (%)	Curing accelerator^4^ (%)	Total NMI (%)
SN	14.71	1.60	0.24	0.05	20.00
CEL	14.18	1.29	0.67	0.46	20.00
OCEL	14.07	1.20	0.87	0.46	20.00
SW	14.21	1.32	0.61	0.46	20.00
OSW	14.25	1.35	0.54	0.46	20.00

1 Formulations: Meat consisted of skinless, boneless breasts. Non-meat ingredients (NMI) were added based on the meat weight. All treatments contained: 1.8 % modified corn starch, 1.2 % dextrose, and 0.40 % sodium tripolyphosphate.

2 Treatments: SN= Sodium Nitrite, CEL= Celery powder, OCEL= Organic Celery powder, SW=Swiss Chard Powder, OSW=Organic Swiss Chard Powder.

3 Added salt content was modified based on the salt content measured in alternative curing powders. Added salt was sodium chloride.

4 Curing accelerator: SN used sodium erythorbate (547 ppm). Alternative cure treatments used cherry powder.

Quality analysis (physicochemical) including cured meat pigments (CMP), total meat pigments (TMP), total myoglobin content (TMC), sodium chloride, and proximate analysis data (moisture, fat, and protein) were analyzed on day 0 with duplicate measurements. Objective color, residual nitrite, and pH were assessed on 15-day intervals from day 0 to day 60 with triplicate measurements. Paired consumer sensory tests and volatile compounds analysis were conducted on day-14 samples to simulate the typical duration products take to reach consumers via the supply chain.

### Product manufacturing

Nitrite concentration was measured for all commercially available vegetable nitrite source curing powders (Veg Stable 506, Veg Stable 531, Veg Stable 532, Florida Food Products, FL, USA; 350001 Diana Foods, La Gare, Antrain, France) before the turkey products were formulated. The concentration of nitrite in the vegetable-based ingredients which included CEL, OCEL, SW, and OSW were determined to be 22,487.0, 17,262.5, 24,531.1, and 27,711.4 ppm, and salt content were determined to be 56, 43, 51, and 61 %, respectively. The curing accelerator was added at 547 ppm sodium erythorbate (Ultrasource LLC. Kansas City, Missouri, USA) for SN and ascorbate equivalent (Veg Stable 515, Florida Food Products, FL, USA) for vegetable source nitrite TRTs. All TRTs were formulated accordingly and manufactured in a random order. Frozen turkey breasts were purchased from a local supplier with a kill date approximately 1 month from the manufacture date, and were thawed in a cooler (1°C) for 5 days before manufacture. Turkey breasts were trimmed of any visible fat, skin, and other defects. Turkey breasts were ground in a commercial meat grinder (Bird Commercial Food-Preparing Machine 538A, The Bird MFG. Co., Marblehead, OH, U.S.A.) using a kidney plate (400 3HK Triumph Kidney Plate, Speco Inc. Schiller Park, IL, U.S.A.). The temperature of the ground turkey breast was checked after grinding to ensure that no excessive temperature elevation had occurred. Ground turkey was subsequently mixed with formulated brine (Table 1) at 20 % percent based on the meat weight. The ground turkey mixture was transferred to vacuum tumblers and tumbled under vacuum continuously for 1 h to achieve adequate protein extraction and free brine pick-up. The ground turkey mixture for each TRT was placed in a plastic lug, covered with a moisture impermeable butcher paper and stored overnight in a temperature-controlled cooler (room temperature, average 1.1°C). The ground turkey mixture was transferred to a rotary vein vacuum- stuffing machine (Model VF616 Vacuum Stuffer, Handtmann Inc., Lake Forest, IL, U.S.A.) and stuffed into collagen casings (Fibran 105.1 mm diameter collagen casing, ViskoTeepak USA, Kenosha, WI). The stuffed casings were then thermal processed using 60.0, 65.6, 71.1, 76.7 and 82.2 °C ramped steam cooked until an internal temperature of 73.8 °C was achieved using a commercial meat smoker (Mauting UKM2001, Mauting s.r.o., Valtice, Czech Republic). Internal temperature was monitored by a calibrated thermal probe inserted into the geographic center of the stuffed casing. After thermal processing, the products were transferred to an RTE area and chilled for 12 h at 1°C. The casing was removed from the stuffed log and the product was sliced into 3 mm and 20 mm slices and vacuum packaged into 4 mil moisture and oxygen impermeable bags (Uline S-19920 Vacuum Bag, Uline Inc., Pleasant Prairie, WI., U.S.A) using a chamber vacuum machine at full strength for 30 sec (Multivac C 500 Double chamber machine, The Multivac Group Kansas City, MO, U.S.A.) for consumer sensory evaluation and chemical analyses, respectively. The slices were stored in an LED lighted (absence of UV-A/B) cooler during a 60-day sampling period (1.1°C) prior to all sensory and physicochemical analyses.

### Proximate composition

Proximate composition was measured on samples from all treatments which included crude protein, fat, and moisture using AOACs procedures. Fat and moisture content in samples were analyzed using a meat analyzer (CEM Smart 6 Meat Analyzer, CEM Co., Matthews, NC, U.S.A.) with an automatic calibration function (AOAC Official Method 2008.06, 2023). Protein content in samples was analyzed by combustion using an elemental analyzer (Leco 828 series, LECO Corporation, St. Joseph, MI, USA). Samples for proximate composition analysis, conducted duplicate, were collected from day 0 for all measurements and kept frozen (−80°C).

### Salt content determination

Salt content of nitrite source vegetable powder was determined by an ion-exchange electrode meter (Horiba LAQUA twin Salt −11; measurement accuracy ± 2 % full scale: 0.0 to 9.9 g/L; Horiba Inc., Kyoto, Japan) according to manufacture instructions. The ion exchange electrode was calibrated with standard solutions (0.5 % and 5.0 %) at 23°C room temperature and 46 % relative air humidity. Nitrite source vegetable powder was diluted in MilliQ water (resistivity 18.2 MΩ·cm @ 25°C) at a ratio of 1:20, followed by filtering through Whatman #1 filter paper. A 100 µL aliquot of the filtrate was transferred to the ion-exchange electrode meter’s flat sensor for measurement. Results were multiplied with the predetermined dilution factor. All measurements were conducted in triplicate.

### Cooking yield

Cooking yield was calculated by weighing the stuffed casing for each treatment prior to thermal processing, and again after overnight stabilization (chilling) prior to slicing (Claus et al., 1990). Subsequently, the casing was peeled from each sample and weighed. Casing weight was subtracted from the initial gross weight to determine the net raw product weight, and from the final gross weight to determine the net cooked product weight.

Cooking yield percentage was then calculated as follows:

$$Cooking\;Yield\;\left(\%\right)=\frac{Net\;cooked\;product\;weight}{Net\;raw\;product\;weight}\times \;100$$

### Lighting display, color, and cured pigment determinations

Color measurement was taken using a handheld portable spectrophotometer (Konica CM-600d, Konica Minolta Inc., Chiyoda, Tokyo, Japan). The colorimeter was calibrated using a white calibration cap (CM-A177, Konica Minolta Inc., Chiyoda, Tokyo, Japan). Measurements (*n* = 5) of white calibration caps were completed automatically with a preinstall program on the spectrophotometer. The device was equipped with illuminant D65, an 8 mm aperture, and a 10 ° standard observer. Color measurements were collected using Commission International de I’Eclairage (CIE) L* (lightness), a* (redness), and b*(yellowness) values.

A light display depletion study was conducted on all treatments (TRTs) to examine how light exposure influenced color fading on deli-style turkey. This study was carried out in a dark room with key access control to ensure consistent conditions. Four fluorescent light tubes (Philips F34T12/835/EW 34 Watts. Philips Inc. Cambridge, MA, U.S.A.) were used as the light source emitting UV-A/B. Fluorescent light tubes were suspended approximately 0.8 meter above the slices of deli-style turkey (3 mm thickness) wrapped by oxygen permeable plastic film (AEP Industries Inc., South Hackensack, N.J. U.S.A. Model: Sealwrap with oxygen transmission rate at 98.4 cm^3^/100 cm^2^/day and water vapor transmission rate at 621 g/m^2^/day at 37.8°C with 100 % Relative Humidity). The UV-A/B intensity was measured by a UV-A/B intensity detector with a handheld sensor (General UV513 A/B, General Tools & Instruments LLC. Secaucus, NJ, U.S.A.) according to manufacture instruction, the average UV energy intensity was measured at 22µw/cm^2^.

Cure color ratio was measured in duplicate using a scanning reflectance spectrophotometer (model UV2600 UV-Vis Spectrophotometer, Shimadzu Inc., Nakagyō-ku, Kyoto, Japan) coupled with a multipurpose large sample component (model UPC-2600, Shimadzu Inc., Nakagyō-ku, Kyoto, Japan). Calibration was conducted with a standard white ceramic calibration tile (L* = 97.06, a* = 1.93, b* = 0.14) wrapped with the same film used for meat color measurement. Cure color ratio (reflectance at 650 nm/570 nm) was used to describe cured color intensity (1.1, no cured color; 1.6, moderate fade; 1.7–2.0, less intense but noticeable cured color and 2.2–2.6, excellent cured color) (King et al., 2023).

### Total and cured meat pigments measurement

Total pigments, cured pigments, and cure efficacy measurements and calculations were determined following AMSA Color Measurement guidelines (Bailey et al., 1964; King et al., 2023). Meat samples (200 g, fully cooked) were minced (model Robot Coupe BLIXER2 Blixer Vertical, Robot Coupe Inc., Vincennes, France) for 10 sec. For nitrosoheme, 10 g of minced sample were weighed into a 100 mL beaker with 43 mL solution containing 40 mL of acetone and 3 mL of MilliQ water. After intermittent mixing of the sample for 5 min, the sample was filtered (Whatman #3, Cytiva Inc., Marlborough M.A., U.S.A) into a 50 mL polyethylene centrifuge tube. A 1.5 mL sample of the filtrate was transferred into a 1-cm quartz cuvette for measuring absorbance at 540 nm. Nitrosoheme content expressed as NO-hematin was calculated based on the formulation: sample A_540_ × 290 with all measurements performed in duplicate. For total heme pigment, 10 g of minced sample were weighed into a 100 mL beaker with acidified acetone (40 mL of acetone, 2 mL of MilliQ water, and 1 mL of 37 % hydrochloric acid). The mixture was stored (1 h) at room temperature and intermittently stirred before filtering (Whatman #3 filter) into a 1-cm quartz cuvette. Optical density was measured at 640 nm to determine total heme content based on the formulation: sample A_640_ × 680. All measurements were duplicated. All steps were conducted in a lab space with only LED lighting that does not emit UV-A/B as confirmed with a handheld UV detector (General UV513 A/B, General Tools & Instruments LLC. Secaucus, NJ, U.S.A.). Curing efficacy was calculated as the percentage of nitrosohemochrome (expressed as ppm acid hematin) divided by total pigments (expressed as ppm acid hematin) times 100.

### pH measurement

pH measurement was conducted with modification according to a method developed by Sheng et al. (2025c). Meat samples (5 g) were blended with a polytron blender (15,000 rpm) with ultrapure water (resistivity of 18.2 MΩ.cm) at a 1:9 ratio. The mixture was then filtered (Whatman #1 filter paper) and measured with a pH meter (model 13-620-AE6 Accumet™; Fisher Scientific Waltham, MA, U.S.A.). Calibration of the pH meter was conducted with NIST certified potassium biophthalate buffer (pH = 4.00) and potassium monobasic and sodium hydroxide buffer (pH = 7.0).

### Residual nitrite and nitrate measurements

Residual NO_2_^−^ and NO_3_^−^ were analyzed using a high-performance liquid chromatography (HPLC) equipment (ENO-20 NOx Analyzer, Eicom Inc, Kyoto, Japan) coupled with a temperature controlled autosampler (AS-700, Amuza Inc., San Diego, C.A., U.S.A.) according to the method described by Sheng et al. (2025c) with modifications. The HPLC analysis for NO_x_^-^ was designed based on the Griess nitrite test adopted by the Association of Official Analytical Chemists (AOAC). Absorption was measured at 540 nm by the UV-Vis detector preinstalled in the nitrite analyzer. Samples (processed meats and meat analogues) were powdered in liquid nitrogen and stored at −80°C until analysis. A 5-gram sample was weighed into 45 mL of pH 7.4 phosphate-buffered saline (PBS), mixed and then split into two equal volumes before being centrifuged at 3500 × *g* at 4°C for 5 min (J6-MI centrifuge equipped with JA-25.50 rotor; Beckman Coulter, Indianapolis, IN, U.S.A.). After centrifugation, 500 μL of supernatant from each slurry and 500 μL of 100 % methanol were mixed, transferred to a 1.5-mL snap cap centrifuge tube, and vortexed at 3000 rpm at room temperature for 10 sec with a digital vortex mixer (cat. no. 0215370, Fisher Scientific, Hanover Park, IL U.S.A). The samples were then centrifuged for 16 min at 15,000 × *g* at 4°C (Eppendorf 5424 centrifuge, Brinkmann Instruments, Westburg, NY, U.S.A.). Supernatants (200 uL) were pipetted into 96-well plates for quantification with the HPLC equipment described above. The HPLC carrier pump speed was set at 40 mL/h and reactor pump speed was set at 13.2 mL/h. A calibration curve was created using 2, 4, 8, 16 ppm of sodium nitrite and sodium nitrate. A sodium nitrite standard (8 ppm) was tested at the start and end of each run. Quantitative data (area under the curve) were analyzed with PowerChrom (version 16.0, New South Wales, Australia).

### Consumer sensory panel

The consumer sensory analysis was conducted in accordance with American Meat Science Association research guidelines for cookery, sensory evaluation, and instrumental tenderness measurements of meat (Association, 2015). Six consumer sensory panels (3 consecutive days for each replication) were conducted at the University of Wisconsin-Madison’s MSABD laboratory (approved by the University of Wisconsin-Madison Institutional Review Board) between day 14 and day 21 post-manufacturing. Consumer panelists who participated in this sensory testing were comprised of university faculty, staff, students, and members of the general public all being at least 18 years of age. Panelists were recruited through the University of Wisconsin-Madison mass email distribution (approximately 88,537 invitations to University of Wisconsin-Madison students, staff, and faculty and UW Health Employee) and an IRB approved poster. Each panelist was served samples in an individual booth that was separated from the sample preparation area through a one-way glass window. Light intensity in each booth was maintained between 1613 and 2152 lux. Light intensity was measured by a handheld spectrometer on every testing day (Sekonic C-700 Spectrometer, Serial # JT12-001230. Sekonic Inc. Nerima-ku, Tokyo, Japan).

Each panelist was served 4 samples randomly selected from the five TRTs. The random selection consisted of all possible combinations containing 10 base designs such that each treatment was sampled equally by panelists. Panelists were served one sample at a time and were provided potable water to cleanse their palate between samples. Deli-style turkey samples were sliced into 0.3-0.35 cm thickness and served cold (3.4-5.2°C) in a sample cup covered with lid.

Consumer liking was measured using a 9-point hedonic index for color, aroma, and overall liking with anchors from “dislike extremely” to “like extremely” with “neither like nor dislike” in the middle of the scale. For cured flavor, non-meat aftertaste, and earthiness, the anchors ranged from none to extremely strong. For bitterness, a 5-point just-about-right scale was used (much too weak to much too strong). Purchase intent was measured using a 5-point scale (definitely not” to “definitely would, may or may not in middle of the scale). Responses from panelists were collected using an iPad (5th generation iPad, OS Version 15.6.1, Apple Inc., Cupertino, CA, USA) with the enabled guided-access mode running in Compusense (Compusense Inc., Guelph, ON, Canada) for data acquisition.

### Volatile compounds analysis

Volatile compounds (VOCs) analysis was conducted according to a method described by Wettasinghe et al. (2001) with modifications. Multiple studies have been conducted on extraction methods and confirmed that steam distillation generally extracts more VOCs than solid phase microextraction methods (Hong et al., 2018; Madruga et al., 2009; Watkins et al., 2012; Xie et al., 2016).

RTE Deli-style turkey samples (200 g) were ground by a commercial meat blender (Robot Coupe BLIXER2 Commercial Blender with a 2.5-Quart stainless steel bowl, Robot Coupe Inc., Vincennes, France). Ground samples (10 g) were then mixed with 10 g of sodium chloride in a 200 mL volumetric tube specifically designed to fit in a fast steam distillation system (SCP DigiPREP Distillation System, SCP Science, Baie-d'urfe, Canada). The distillation was conducted at 60 % strength for 300 sec. Distillate (100 mL) was mixed with 150 mL of methylene chloride in a 500 mL separatory flask and mixed vigorously and stored at ambient temperature (23°C) for 2 h. The methylene chloride layer was vaporized under vacuum using a rotary evaporator (Rotavapor, Buchi, Flawil, Switzerland) at 39.6°C. When the contents reached a volume of approximately 5 mL, they were carefully removed from the rotary evaporator and mixed with 5 g of ammonium formate to remove any water in the solution. The concentrate then was transferred into a dark glass vial and stored at −80°C until analysis.

Gas chromatography mass spectrometry tandem (GC-MS/MS) analysis was conducted using a GC-MS/MS system coupled with an autosampler (Shimadzu GCMS-TQ 8040NX with AOC-20 plus autosampler Shimadzu Inc., Nakagyo-ku, Kyoto, Japan) supplied with helium gas and a smart switch that utilized nitrogen gas in the savor mode. Separation of VOCs was conducted on a general-purpose fused silica low polarity, crosslinked diphenyl dimethyl polysiloxane phase column (Shimadzu SH-I-5MS Capillary Column, 30 m x 0.25 mm x 0.25um, Shimadzu Inc., Nakagyo-ku, Kyoto, Japan) Oven temperature was programed from 45°C to 240°C at a rate of 5°C/min with an initial and final hold time of 5 and 10 min, respectively. The total run time was 60 minutes. For the mass spectrometry detector, the electron ionization energy was set at 70 eV. The mass range, electron multiplier voltage, and scan rate were set at m/z 33-330, 2000 V, and 20,000 u/sec, respectively. Ionization source temperature was maintained at 230°C (Li et al., 2023).

VOCs were identified by matching mass spectral data of sample compounds with an Electron Spray (EI) NIST database (**N**IST 23 Tandem Mass Spectral Libraries). The area under the curve (AUC) was integrated using Savitzky-Golay methods with width, setting at 0.24 sec. Each integrated area was compared with the EI database based on spectrum similarity then manually analyzed based on fragmentation patterns. The results of each sample TRT were integrated for comparison using Python (Python version 3.12.7, The Python Software Foundation) on Spyder (The Scientific Python Development Environment, version 6.0.1, Spyder-IDE.org) as an Integrated Development Environment (IDE).

### Statistical analysis

The main factorial design was a split plot for measurement over time (day of sampling). The experiment model included the main effects of treatments, day of sampling, and the interaction of treatment × day. A two-way analysis of variance (ANOVA) was conducted to evaluate the main effects of source of nitrite and time (day of sampling), as well as their interaction effect on color (CIE L*, a*, b*, Hue angle, and Chroma C*), pH and residual nitrite (R version 4.4.1, http://www.r-project.org) with the standard installed packages. One-way ANOVA was conducted to evaluate the effects of source of nitrite on proximate analysis (moisture, fat, protein), salt content, cooking loss, and meat pigment analysis (CMP, TMP, cure efficiency, and TMC). Pearson correlation and principal component analysis (PCA) were used to analyze sensory attributes and volatile compounds. Correlation coefficient (“r”) was used to determine positive or negative relationships. PCA dimensionally reduced all variables into two principal components. Principal components PC1 and PC2 were used to describe data relationships based on eigenvalues (from parallel analysis).

## Results and discussion

### Proximate composition

Proximate analysis results of cooked deli-style turkey cured with various sources of nitrite (day 0 samples) are depicted in Table 2. All compositions (moisture, protein, fat, and salt) of RTE deli-style turkey cured with either conventional or organic vegetable sources of nitrite were similar to that of the control made with SN. Furthermore, there were no differences (*P* > 0.05) in moisture, fat, and protein content among the alternative cure treatments. The proximate composition values were in alignment with previous studies that had reported using similar formulations (Lee and Ahn, 2005). Salt concentrations in the cooked deli-style turkey ranged from 1.49 to 1.54 %, but no treatment differences (*P* > 0.05) were found. Cooking yields ranged from 92.4 to 93.7 % but no differences (*P* > 0.05, data not shown) were found among TRTs. Cooking yields were lower than previous studies, potentially due to the higher cooking temperature (internal temperature reached 73.98°C) and greater added water than a previous study (Grasso et al., 2016; Muriana et al., 2004).

**Table 2:** Least square means of proximate analysis of all Deli-style turkey TRTs.

Treatment^1^	SN (%)	CEL (%)	OCEL (%)	SW (%)	OSW (%)	SEM^2^ (%)
Moisture	71.72^a^	71.82^a^	71.57^a^	71.85^a^	71.77^a^	0.23
Protein	23.93^a^	23.95^a^	23.97^a^	23.91^a^	23.96^a^	0.06
Fat	1.45^a^	1.43^a^	1.47^a^	1.45^a^	1.41^a^	0.03
Salt^3^	1.51^a^	1.54^a^	1.49^a^	1.53^a^	1.52^a^	0.02

1 The treatment abbreviation refers to nitrite source (sodium nitrite, SN; Celery powder, CEL; Organic Celery powder, OCEL; Swiss Chard Powder, SW; Organic Swiss Chard Powder; OSW).

2 SEM = standard error of means. *n* = 20. Means with unlike superscript letters within the same row denote differences (*p* < 0.05).

3 Salt content was expressed as sodium chloride equivalent.

The sodium content of deli-style turkey is a crucial determinant of its sensory attributes, where excessive amounts may result in diminished sensory perception (Pandya et al., 2020; Schroeder, 2013). Excessive salt can mask other flavors, making food taste one-dimensional and unbalanced. It can also overwhelm the taste buds, resulting in an overpowering salty taste (Paulsen et al., 2012; Wang et al., 2019). A previous study conducted on sensory attributes of alternative cured turkey did not include the effect of salt content from the vegetable powder on finished products (Redfield and Sullivan, 2015). In contrast in our study, we standardized both nitrite and salt content in the brine that was incorporated into the turkey meat by accounting for the sodium content in the pre-converted vegetable powder. The salt content in pre-converted vegetable powders ranged from 46 to 64 %. With a typical formulation of processed meats containing 156 ppm sodium nitrite equivalent from vegetable powder, a 0.25 to 0.31 % variation in salt content could result in the brine if the salt content was not standardized.

### Residual nitrite and cure efficiency

**Fig. 1:**
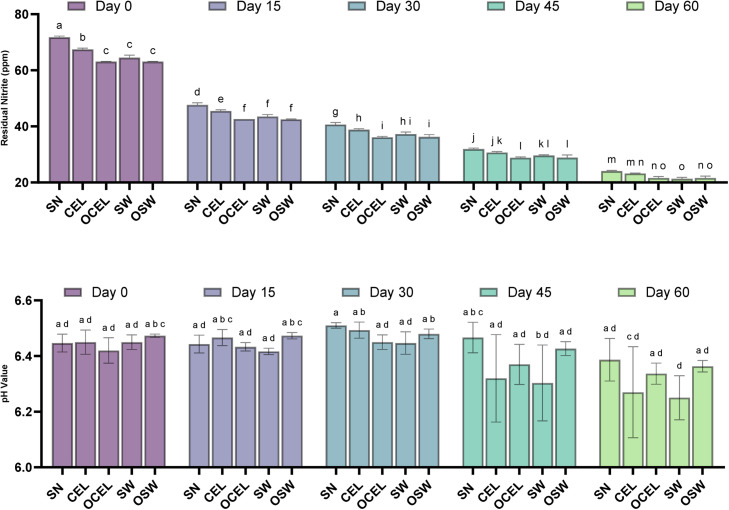
Residual nitrite content of all RTE deli-style turkey TRTs over a storage time of 60 days. Treatments: SN = Sodium Nitrite; CEL= Celery powder; OCEL=Organic celery powder; SW= Swiss chard powder; OSW = Organic Swiss chard. Unlike letters denote a difference (*p* < 0.05). Error bars represent standard errors.

Residual nitrite (NO_2_^-^) in SN and all other treatments decreased 59.1 to 72.7 % from day 0 to day 60 (Fig. 1). Residual nitrite in SN was significantly higher (*P* < 0.05) than all alternative cure treatments from day 0 to day 30. The same finding was reported by a previous study on natural and organic meat curing processes (Sebranek et al., 2012). This is possibly due to plant polyphenols in alternative cure powder that simulate NO_2_^-^ reduction to nitric oxide (NO^-^) during processing (Hernández-Ramírez et al., 2009; Schroeder, 2013; Weitzberg and Lundberg, 2013). Conventional nitrite source treatments contain less polyphenols (CEL and SW) measured generally higher NO_2_^-^ than that of the organic nitrite source (OCEL and OSW) on day 0 (data not shown). These results aligned with previous reports that SN products contain more NO_2_^-^ than vegetable pre-converted nitrite source treatments (Redfield and Sullivan, 2015). Thermal processing decreased 51.0 to 53.2 % of the ingoing nitrite which supports previous studies using similar formulations of deli-turkey (Myers et al., 2013; Redfield and Sullivan, 2015). During storage, the most noticeable decrease of NO_2_^-^ occurred on day 15 after production with an average decrease of 38 % among all treatments.

The pH values in processed meats were regarded as one of the most influential factors affecting the interconversion of NO_2_^-^, nitrate (NO_3_^-^), and nitric oxide (NO) (Sheng et al., 2025c). Lower pH can lead to a more rapid conversion from NO_3_^-^ to NO_2_^-^, and NO_2_^-^ will be reduced and then degraded into NO under acidic conditions. Conversion of NO_2_^-^ to NO^-^ may become accelerated when the pH is less than 6 (Jo et al., 2020). In this study, we found that the pH value of all TRTs gradually decreased during storage (Fig. 1) from a mean value of 6.46 on day 0 to a mean value of 6.31 on day 60, with no significant differences among the treatments (*P* > 0.05).

**Fig. 2:**
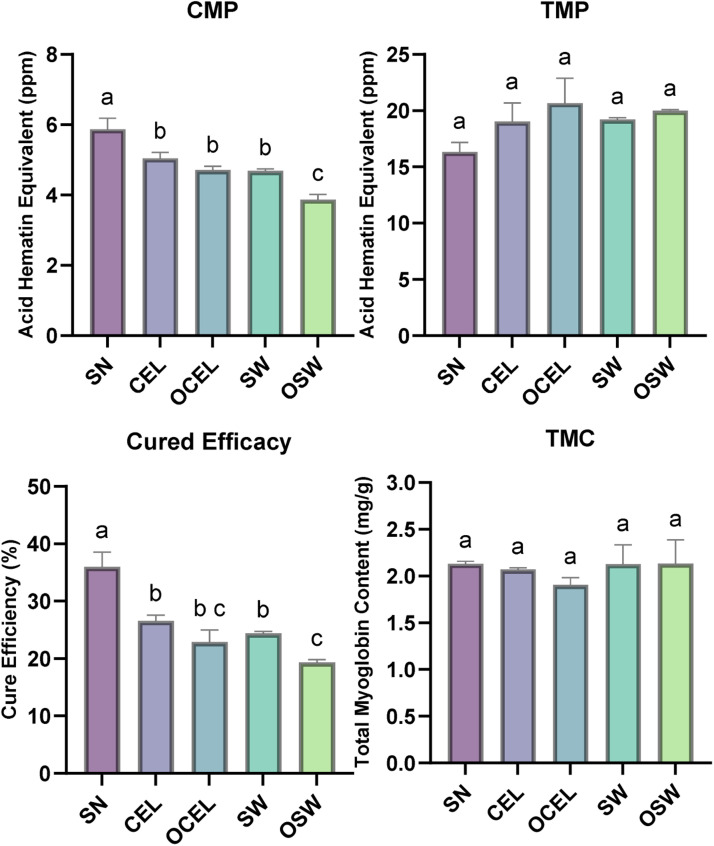
Physicochemical analysis on cooked deli-style turkey products Treatments: SN = Sodium Nitrite; CEL= Celery powder; OCEL=Organic celery powder; SW= Swiss chard powder; OSW = Organic Swiss chard. Dependent variables: CMP = Cured meat pigments; TMP = Total meat pigments; Cured efficiency = Cured meat pigments/Total meat pigments; TMC= Total Myoglobin content. Unlike letters within a dependent variable denote a difference (*p* < 0.05). Error bars represent standard errors.

Another method utilized in this study to understand meat color chemistry was cured meat pigment conversion analysis (Fig. 2). Cure efficacy of SN was higher than the rest of vegetable source nitrite treatments (*P* < 0.01). There were no differences (*p* < 0.05) in TMP and TMC among all treatments while the CMP was significantly higher in SN and lower in OSW. The organic source of nitrite has been observed to have a lower cure efficiency compared to its conventional counterpart. This reduction in cure efficiency is potentially due to the interaction between polyphenols and nitrite in the organic vegetable powder (Luo et al., 2021) leading to less available nitrite to react with meat color pigments.

### Objective color

**Fig. 3:**
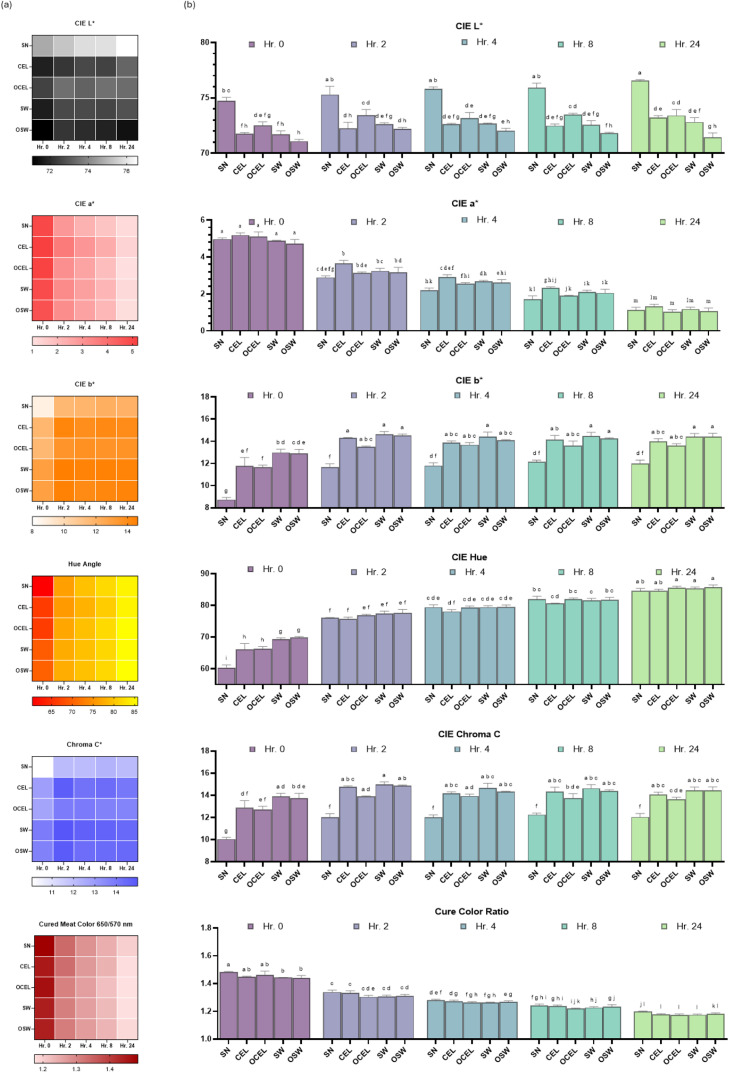
Display depletion study on deli-style turkey TRTs in 24 hours display study under UV A/B exposure from fluorescent light. (a) Trend of CIE L*, Cured meat color ratio at 650/570 nm, Hue Angle, and Chroma C* (b) Bar plot of CIE L*, Hue Angle, and Chroma C*. Treatments: SN = Sodium Nitrite; CEL= Celery powder; OCEL=Organic celery powder; SW= Swiss chard powder; OSW = Organic Swiss chard. Unlike letters with a dependent color variable denote differences (*p* < 0.05). Error bars represent standard errors.

Color is one of the most important characteristics that influence consumer’s perception of sensory attributes towards processed meats (de Araújo et al., 2022). In this study, we evaluated the objective color of the treatments (TRTs) using two methods: storage in a dark environment and exposure to fluorescent light. The color change patterns in dark storage and under fluorescent light were very similar, as shown by the color depletion trends displayed in Fig. 3.

CIE L* indicates lightness, with values ranging from 0 (black) to 100 (white). SN remained lighter (*p* < 0.05) than alternative cures throughout the 24 h display. Based on the vegetable source, the organic alternative cures were not different (*p* > 0.05) than the non-organic alternative cures with the exception that SW was lighter than OSW at 24 h.

CIE a* indicates the position between red/magenta and green, with positive values indicating red/magenta and negative values indicating green. The redness of SN is similar (*p* > 0.05) to alternative cures except (*p* < 0.05) lower than that of CEL at 2 and 4 h There are no differences been observed based on the vegetable sources with the exception that CEL has significantly higher (*p* < 0.05) redness value than OSW at 4 h.

CIE b* indicates the position between yellow and blue, with positive values indicating yellow and negative values indicating blue (King et al., 2023). SN has a significantly lower (*p* < 0.05) yellowness value than alternative cures during display. The organic alternative cures were not different (*P* > 0.05) from the non-organic alternative cures except at 0 h, where OCEL had a significantly lower CIE b* value than the Swiss chard treatments.

Hue Angle represents the overall color perceived by the human eye, calculated as an angle in a color wheel (Seyfert et al., 2004). At 0 h, SN had a smaller hue angle (*P* < 0.05) than the alternative cures during display. Based on the vegetable source, the organic alternative cures were not different from the non-organic alternative cures. Among different vegetable cures, the celery TRTs had a lower (*P* < 0.05) hue angle than the Swiss chard TRTs at 0 h possibly due to a lower yellowness previously discussed.

Chroma C* indicates color saturation, with higher values representing more vivid colors (King et al., 2023). SN resulted in less color saturation (*P* < 0.05) than the alternative cures during display. Based on the vegetable source, the organic alternative cures were not different from the non-organic alternative cures. Among different vegetable cures, Celery treatments (TRTs) had a lower (*p* < 0.05) saturation than Swiss chard TRTs at 0 h, possibly due to a lower yellowness and hue angle.

Cured Color Ratio (Reflectance ratios 650/570 nm) is used to determine differences in cured meat color intensity (Seyfert et al., 2004). SN exhibited greater nitrosohemochrome than SW and OSW at 0 h However, no differences (*P* > 0.05) were found between SN and the alternative cures during the remainder of the display. No differences were observed based on organic and non-organic sources or the species of vegetable used.

Overall, SN has lower yellowness, hue angle, and saturation than the alternative cured treatments (TRTs). This finding aligns with a similar previous study on deli turkey (Redfield and Sullivan, 2015). Based on the vegetable sources, celery TRTs have lower yellowness, hue angle, and saturation than Swiss chard. The objective color differences among treatments become not noticeable at 24 h, except for a lower yellowness in SN. The differences in objective color in the TRTs were potentially due to the plant pigments present in the vegetable nitrite source powder. Celery contains known photosynthetic pigments such as chlorophyll, carotenoids, and anthocyanins (Li et al., 2022). Swiss chard is rich in chlorophylls, carotenoids, betalains, betacyanins, and lycopenes (Barickman and Kopsell, 2016). Cherries relative to the use of cherry powder as the curing accelerator in the current project, contain anthocyanidins (Li and Wagenknecht, 1958). Anthocyanidins are a class of water-soluble pigments that significantly contribute to the color of foods, imparting red, purple, and blue hues to many vegetables (Mattioli et al., 2020). Furthermore, even though limited objective color differences were observed between organic versus non organic TRTs, vegetable produced with organic practices may exhibit higher nutritional quality concerning photosynthetic pigments compared to conventionally grown counterparts (Fernanda et al., 2016). Thus, when organic produced vegetable curing powders are added into meat as food ingredients, they may function differently than their conventional grown counterparts.

### Sensory evaluation

**Fig. 4:**
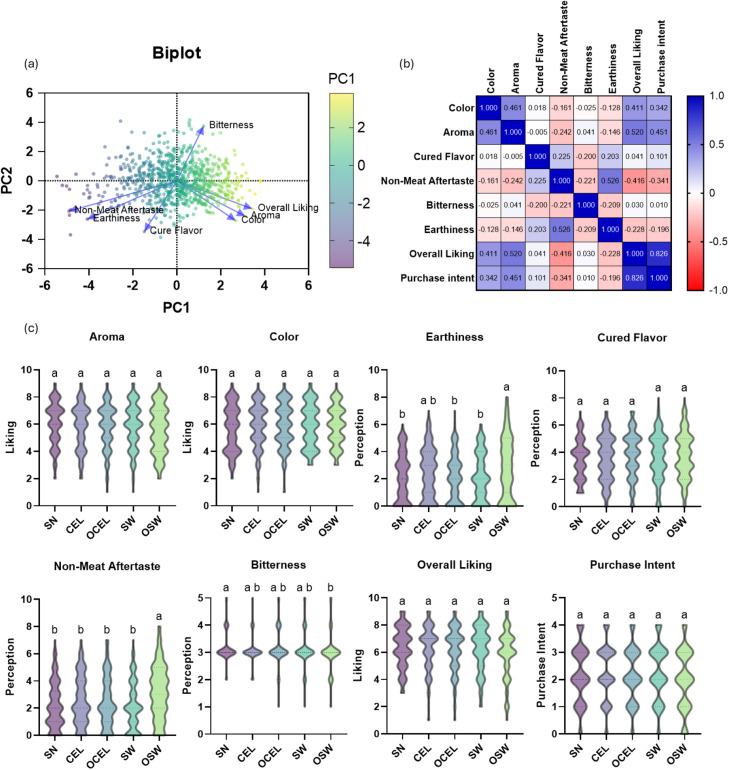
Sensory attributes analysis of all Deli Turkey TRTs. (a) Biplot of Deli-Turkey sensory attributes using PCA Analysis; (b) Correlation Analysis of all sensory attributes of Deli-turkey; (c) Violin plot of sensory attributes of all TRTs. In Fig. c, unlike letters denote difference (*p* < 0.05). Treatments: SN = Sodium Nitrite; CEL= Celery powder; OCEL=Organic celery powder; SW= Swiss chard powder; OSW = Organic Swiss chard. Sensory scales: aroma, color, and overall liking 9-point hedonic (dislike extremely to like extremely). For earthiness, cured flavor, and non-meat aftertaste 9-point scale (none to extremely strong). For bitterness, a just-about-right 5-point scale (much too weak to much too strong). Purchase intent 5-point scale (definitely not to definitely would). Unlike letters with a dependent sensory trait denote differences (*p* < 0.05). Error bars represent standard errors.

Bitterness, non-meat aftertaste, and earthiness were found to be negatively correlated with overall liking using principal component analysis (PCA) (Fig. 4a). Pearson correlation (Fig. 4b) confirmed this observation and further indicated that aroma and color were moderately positively associated with overall liking (r ranges from 0.41 to 0.52) and purchase intent (r ranges from 0.34 to 0.45), while earthiness and non-meat aftertaste were negatively associated with overall liking (r ranges from −0.20 to −0.23) and purchase intent (r ranges from −0.34 to −0.42). No differences (*P* > 0.05) were evident in consumer acceptability among all treatments regarding to aroma, color, cured meat flavor, overall liking, and purchase intent (Fig. 4c). Significantly more earthiness (*P* = 0.026), less bitterness (*P* = 0.09), and non-meat after taste (*P* < 0.001) were perceived from OSW treated products than the remaining TRTs. There were no differences among organic source nitrite cured products and conventional source nitrite cured products. A previous study concluded that sodium nitrite cured products had better color acceptability than products made with SN (Redfield and Sullivan, 2015).

### Volatile compounds analysis

**Fig. 5:**
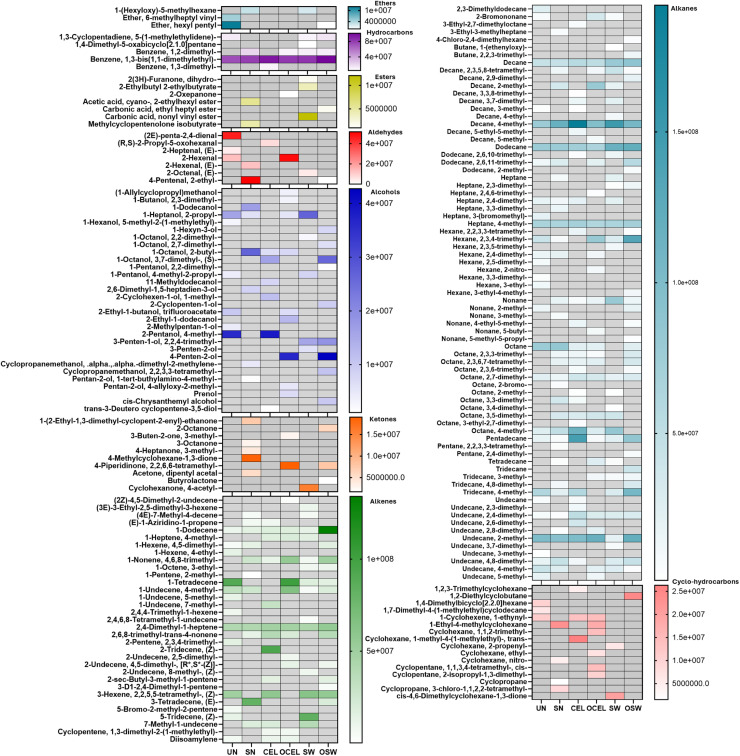
Complete volatile compounds (VOCs) comparison of all treatments of deli-style turkey (value expressed in relative area under the curve).UN= uncured reference. Treatments: SN = Sodium Nitrite; CEL= Celery powder; OCEL=Organic celery powder; SW= Swiss chard powder; OSW = Organic Swiss chard. Grids in gray color indicate data not applicable.

**Fig. 6:**
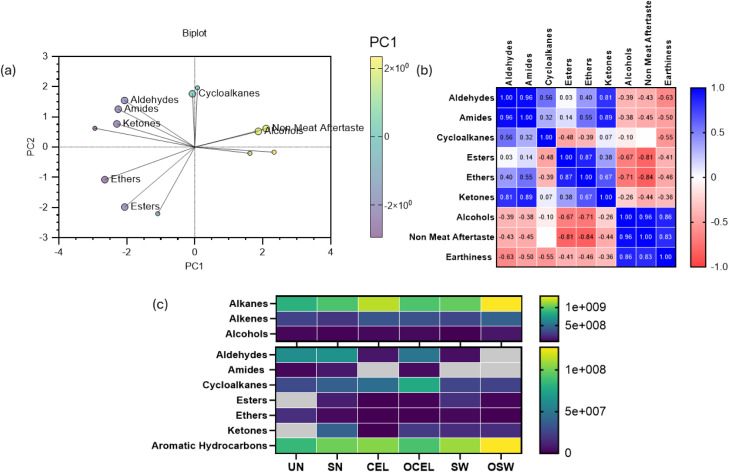
Volatile compounds of RTE deli-style turkey cured with different sources of nitrite. (a) Biplot of deli-style turkey VOCs and negative sensory attributes. (b) Correlation analysis of deli-style turkey VOCs and negative sensory attributes. (c) Key volatile compounds relative concentration in all treatments of deli-style turkey. Treatments: SN = Sodium Nitrite; CEL= Celery powder; OCEL=Organic celery powder; SW= Swiss chard powder; OSW = Organic Swiss chard. Grids in gray color indicate data not applicable.

A total of 586 volatile organic compounds (VOCs) were identified (Fig. 5). Among these, 196 compounds yielded a spectrum similarity match above 80 % compared with the current National Institute of Standards and Technology (NIST) libraries. The principal component analysis (PCA) analysis in Fig. 6(a) indicated that non-meat aftertaste was strongly positively associated with alcohols. Pearson correlation analysis in Fig. 6(b) confirmed this observation and also indicated a strong positive correlation between earthiness and non-meat after taste (*r* = 0.53). Color and aroma were also found to be positively correlated (*r* = 0.46). Non-meat aftertaste was identified as negatively correlated with overall liking (*r* = −0.42) and purchase intent (−0.34). Earthiness was slightly negatively correlated with overall liking (*r* = −0.23) and purchase intent (*r* = −0.20). Non-meat aftertaste and earthiness also slightly negatively correlated with Color and Aroma perception (*r* = −0.13 to −0.24). Furthermore, aromatic hydrocarbons were found to be most abundant in OSW (Fig. 6c). Overall aldehyde content in organic treatments was found to be lower than in conventional TRTs. It is worth noting that besides volatile compounds, the color of many foods and beverages influences the perception of their aroma by shaping expectations. Visual cues are the first sensory impression perceived by consumer panelists (Cardello, 1994; Garber Jr et al., 2000).

In Fig. 5, a complete heat map illustrates the distribution of all identified compounds with relative concentration calculated by AUC. A distinctively different VOCs profile was found for deli-turkey cured with different methods. Most aromatic VOCs are formed through two mechanisms, lipid oxidation and Maillard reaction (Diez-Simon et al., 2019; Sun et al., 2022). Thermal processing is known to increase lipid oxidation (Toldrá et al., 2007). Turkey has been shown to contain fewer VOCs than many other common species of meat products (Yang et al., 2022).

Hydrocarbons, including alkanes and alkenes, were widely identified in deli turkey control and treatments. Alkanes and alkenes have been considered as part of the lipid oxidation products (Oyerinde et al., 2023). In this study, alkanes were the most abundant VOCs identified, followed by alkenes. Specifically, decanes, dodecanes, heptanes, pentadecanes, and undecanes were found in both control and treatment groups. Short-chain alkanes may contribute to the aromas associated with fat and greasy smells. Overall, alkanes and alkenes impart a light gasoline-like odor to meat products. (Bleicher et al., 2022).

Ketones may be derived from fatty acids oxidation and contributes to buttery aroma note of cooked meats (Peterson et al., 2006). Overall organic treatments (OCEL and OSW) contain less ketones than those of non-organic source ones (CEL and SW). No ketone compounds have been identified in uncured treatment (UN) and CEL treatments.

Aldehydes have a green, grassy taste and a meaty, tallowy order (Shahidi and Hossain, 2022). Aldehydes are also considered a key indicator of lipid oxidation, as they are secondary metabolism products formed during oxidation of unsaturated lipids in meat products (Shahidi, 2001). Hexanals, pentanals, octanal, 2-propyl-5-oxoheptanal, and 2-penta-2,4-dienal were identified in various sample treatments. Notably, 2-hexanal was found at a relatively high content in OCEL, whereas minimal aldehyde content was observed in other alternative cure treatments. This discrepancy is potentially due to the deodorization process applied to the OCEL used in this study, which may have reduced its antioxidant capacity to some extent.

Alcohol chemicals contribute to the flavor of cooked meat and alcohols in cooked meat are primarily produced from lipid oxidation. Alcohols appeared to strongly correlate with non-meat aftertaste (Fig. 5a). 4-penten-2-ol is a member of the fragrance structural group branched chain saturated alcohol found highly concentrated in the OSW treatments that are known to contribute to a nutty or pungent flavor (Nijssen et al., 2009). 3,7-dimethyl-1-Octanol, also known as Citronellol, occurred most abundantly in the OSW treatments while it was not identified in the conventional nitrite source treatment (SW). cis-Chrysanthemal alcohol is a terpene alcohol that might exhibit slight fresh floral scent and was identified in OSW sample but absent from the other TRTs.

## Declaration of competing interest

The authors declare no conflict of interests with this research.

## Appendix Supplementary materials

Image, application 1
