# Supplementary material for: Organic and conventional alternative curing ingredients effects on quality and sensory attributes of deli-style Turkey
Source: Poult Sci. 2025 May 28;104(8):105370. doi: 10.1016/j.psj.2025.105370 (PMC12163148; doi:10.1016/j.psj.2025.105370)
Supplement: Supplementary file 1 [file mmc1.pdf]

EVALUATION OF CURED Deli-Turkey

Please read the informed consent form and, if you agree to participate, sign and date it and pass it through the door at the front of the booth. An attendant will give you your first sample. Please answer all questions. Your name is not on the questionnaire and will not be identified with your answers.

What is your gender?

- ○ Male
- ○ Female
- ○ Prefer not to say

What is your age?

- ○ 18-24
- ○ 25-34
- ○ 35-44
- ○ 45-54
- ○ 55-64
- ○ >64

Which statement most accurately describes how often you typically consume Deli-Turkey?

- ○ several times a week
- ○ once a week
- ○ several times a month
- ○ once a month
- ○ Less than once a month
- ○ several times a year
- ○ rarely
- ○ never

**** Please slide yellow card underneath window to indicate you are ready for next sample****

Please Indicate the 3-Digit Code Number on the Sample ____

You will be asked to rank all samples from most like to least like by the end of the test.

Please rinse your mouth with some water between samples.

How much do you like or dislike the COLOR of the Deli Style Turkey?

Look at the sample and please select your likeness to the COLOR of the sample.

Dislike Extremely	Diskliek Very Much	Dislike Moderately	Dislike Slightly	Neither Like Nor Dislike	Like Slightly	Like Moderately	Like Very Much	Like Extremely

How much do you like or dislike the AROMA of the Deli Style Turkey?

Smell the sample and please select your likeness to the AROMA of the sample.

Dislike Extremely	Diskliek Very Much	Dislike Moderately	Dislike Slightly	Neither Like Nor Dislike	Like Slightly	Like Moderately	Like Very Much	Like Extremely

Please select any cured flavor level

None	Extremely Weak/Little	Very Weak/Little	Modertately Weak/Little	Slightly Weak/Little	Slightly Strong	Moderately Strong	Very Strong	Extremely Strong

How much non-meat aftertaste you perceived of the Deli Style Turkey?

Please select any **non-meat** aftertaste level

None	Extremely Weak/Little	Very Weak/Little	Modertately Weak/Little	Slightly Weak/Little	Slightly Strong	Moderately Strong	Very Strong	Extremely Strong

Taste the sample then please select your likeness to the Bitterness of the sample.

Much Too Weak	Somewhat Too Weak	Just About Right	Somewhat Too Strong	Much Too Strong

Please select any Earthiness aftertaste level

None	Extremely Weak/Little	Very Weak/Little	Modertately Weak/Little	Slightly Weak/Little	Slightly Strong	Moderately Strong	Very Strong	Extremely Strong

**** Please slide yellow card underneath window to indicate you are ready for next sample****

**** Please help yourself to a candy bar/soda after you finish all samples****

OVERALL LIKING of the Deli Style Turkey.

Dislike Extremely	Diskliek Very Much	Dislike Moderately	Dislike Slightly	Neither Like Nor Dislike	Like Slightly	Like Moderately	Like Very Much	Like Extremely

Would you purchase this Deli Style Turkey product?

Definitely will not buy	Probably will not buy	Might or might not buy	Probably will buy	Definitely will buy

1. Please rank your liking of the samples from least liked (left) to most liked (right)

2. Please drag the sample number boxes to the ranking boxes (you may redo this by dragging the sample number boxes back into the light blue area)

1.	2.	3.	4.

BC 1 BC 2 BC 3 BC 4

**** Please slide yellow card underneath window to indicate you are ready for next sample****

**** Please help yourself to a candy bar/soda after you finish all samples****

THE UNIVERSITY of WISCONSIN MADISON

Minimal Risk Research IRB
4/12/2024

Submission ID number: 2024-0540
Title: Alternative Cured Deli-Style Turkey Sensory Evluation
Principal Investigator: Jim Claus
Point-of-contact: Steven Sheng
IRB Staff Reviewer: Laura Conger

The MRR IRB conducted a review of the above referenced initial application. The study was determined to meet the criteria for exempt human subjects in accordance with the following category(ies) as defined under 45 CFR 46:

(2)(i) Tests, surveys, interviews, or observation (non-identifiable)
(6) Taste and food quality

If this study falls under VA regulations, you must get final approval from the VA Research & Development Committee prior to starting research activities.

NOTE: If the research under this exemption application becomes subject to FDA regulations, or other changes are made that could affect the exemption status, you must contact the IRB as the IRB's exemption determination may no longer apply.

You have identified the following financial sources to support the research activities in this IRB application:

- Silva,Erin - ORGANIC ALTERNATIVES TO CONVENTIONAL CELERY POWDER AS A - USDA, NATL INSTITUTE FOOD & AGRICULTURE - 2019-51300-30243

If this information is incorrect, please submit a change to modify your application as appropriate.

To access the materials the IRB reviewed and accepted as part of the exemption determination, please log in to your ARROW account and view the documents tab in the submission's workspace.

Although the human subjects research described in the ARROW application referenced above was determined to meet the federal criteria for exemption and thus does not require continuing review, please be aware of your responsibilities related to the conduct of the research and when additional IRB review is required. Prior to starting research activities, please review the

Principal Investigator and Study Team Responsibilities in the Investigator Manual, which includes a description of the types of changes that must be submitted to ensure the research continues to comply with the conditions of the exemption and/or category(ies) of exemption.

If you have general questions, please contact the Minimal Risk Research IRB at 608-263-2362. For questions related to this submission, contact the assigned staff reviewer.
